# Prognostic signature based on m6A-related lncRNAs to predict overall survival in pancreatic ductal adenocarcinoma

**DOI:** 10.1038/s41598-022-07112-8

**Published:** 2022-02-23

**Authors:** Qiong Wu, Lei Chen, Dongliu Miao, Yiqi Jin, Zhigang Zhu

**Affiliations:** Department of Intervention and Vascular Surgery, Suzhou Municipal Hospital, Affiliated Suzhou Hospital of Nanjing Medical University, Suzhou Cancer Medical Center, No.16, Baita West Road, Suzhou, 215001 Jiangsu China

**Keywords:** Cancer microenvironment, Pancreatic cancer, Cancer models, Tumour immunology, Prognostic markers, Epigenomics

## Abstract

A growing body of evidence indicates that N6-methyladenosine (m6A) and long non-coding RNAs (lncRNAs) play crucial roles in the progression of PDAC and the treatment response of patients with pancreatic ductal adenocarcinoma (PDAC). In this study, we identified m6A-related lncRNAs to reveal their association with PDAC in prognosis and tumor immune environment. A prognostic signature based on 9 m6A-related lncRNAs was established, and the high-risk patients exhibited a significantly worse prognosis than low-risk patients. The predictive capacity was confirmed by receiver operating characteristic (ROC) curve analysis and an independent validation cohort. Correlation analyses revealed that m6A-related lncRNA signature was significantly associated with the number of somatic mutations, immunocyte infiltration, immune function, immune checkpoints, tumor microenvironment (TME) score, and sensitivity to chemotherapeutic drugs. Consequently, we constructed a highly accurate nomogram for improving clinical applicability of signature and exhibited superior predictive accuracy than both the signature and tumor stage. In conclusion, our proposed m6A-related lncRNA signature is a potential indicator predictive of prognosis and immunotherapeutic responses in PDAC patients.

## Introduction

Pancreatic ductal adenocarcinoma (PDAC) remains a lethal malignancy with a dismal prognosis^1^. Despite its low incidence, PDAC represents the fourth leading cause of cancer-related deaths in the United States, but may have moved to second place by 2030 after lung cancer and surpassing colorectal and breast cancers, and its mortality is increasing for both genders^2^. PDAC is typically asymptomatic and the majority of PDAC patients were diagnosed at an advanced stage^3^. Additionally, 60–80% of patients presenting resectable pancreatic tumors will exhibit recurrence, regardless of administration of adjuvant therapy^4^. For patients with an advanced stage of PDAC, neoadjuvant therapy, radiotherapy, chemotherapy, targeted molecular therapy, and immunotherapy have only yielded modest improvements in survival^1^. Therefore, it is significant and urgent to develop dependable biomarkers for accurate risk stratification and prognostic prediction, thereby facilitating individualized treatment and prolonging survival of patients with PDAC.

N6-methyladenosine (m6A), a reversible and abundant modification on messenger RNAs (mRNAs) and non-coding RNAs (ncRNAs), has been demonstrated to greatly affect various aspects of RNA metabolism, including splicing, stability, nuclear export, and translation^5,6^. Several studies have indicated that the aberrant expression of m6A regulators, which include the “writers” (methyltransferases), “readers” (binding proteins), and “erasers” (demethylases), can potentially induce m6A to actively participate in carcinogenesis, cancer development, and drug resistance, including PDAC^7,8^. For instance, high expression of METTL3, an m6A methyltransferase, has been reported to be able to facilitate the proliferation and progression of colorectal cancer, gastric cancer, and PDAC cells^9–11^.

Long noncoding RNA (lnRNA) refers to a class of non-protein coding transcript, which constitute approximately eighty percent of the human whole gene transcriptome^12^. It contains more than 200 nucleotides, which are not translated into proteins, can broadly be classified into: sense, antisense, bidirectional, intronic and intergenic lncRNAs^12^. However, increasing evidence have revealed the contributions of lncRNA in cancer phenotypes by physically interacting with proteins, DNA, and other RNA^12^. Previous studies have uncovered the influence of lncRNAs on the regulation of various biological processes, including tumorigenesis and immunity^13,14^. Mounting evidence supports the notion that the interaction between m6A and lncRNAs is involved in the growth and development of cancer, including pancreatic cancer^15^. For example, He et al.^16^ revealed that the m6A eraser ALKBH5 inhibited pancreatic cancer motility by demethylating lncRNA KCNK15-AS1. IGF2BP2 acts as an m6A reader to up-regulate the expression of lncRNA DANCR, which promotes cancer stemness-like properties and the pathogenesis of pancreatic cancer^17^. Another recent report demonstrated that m6A-mediated up-regulation of lncRNA LIFR-AS1 promotes the progression of pancreatic cancer via miRNA-150-5p/VEGFA/Akt signaling^18^. Thus, an m6A-related lncRNA-based prognostic model may be helpful in the understanding and management of PDAC.

Herein, by utilizing the RNA-sequencing (RNA-seq) data and corresponding clinical information from The Cancer Genome Atlas (TCGA) and International Cancer Genome Consortium (ICGC) databases, we investigated the prognostic and immunologic significance of m6A-related lncRNAs and developed an m6A-related lncRNA prognostic signature to predict survival outcomes in patients with PDAC. The effectiveness and stability of the signature were verified. Meanwhile, we also investigated the correlations of the prognostic signature with clinical features, tumor immune microenvironment, somatic mutation landscapes, and chemosensitivity. Ultimately, nomograms were constructed and allowed for improved accuracy in survival estimation.

## Materials and methods

### Data collection and m6A-related lncRNAs identification

Transcriptome profiling converted into fragments perkilobase million (FPKM) along with clinical data of 178 cases, and the somatic mutation data were downloaded from TCGA database (https://portal.gdc.cancer.gov/repository). GTF file was downloaded from GENCODE (https://www.gencodegenes.org) as annotation to differentiate mRNA and lncRNA. We excluded patient whose follow-up time was less than 30 days. One hundred and seventy patients in total with gene expression data were included. To validate the signature, we downloaded expression data (*n* = 82) from ICGC database (https://icgc.org/). In the present study, TCGA dataset and ICGC dataset were used for training and validation, respectively. The expression matrices of 23 m6A-related genes were obtained based on the latest publications. m6A-related lncRNAs were extracted by co-expression strategy, whose correlation coefficients were over 0.4 and *P*-value < 0.001.

### Establishment of m6A-related lncRNA signature

The TCGA and ICGC cohorts were treated as training and validation sets, respectively. The prognostic signature based on m6A-related lncRNAs was constructed in three steps as follows: (1) univariate Cox regression analysis was conducted to screen m6A-related lncRNAs that significantly correlated with the overall survival (OS) of PADC patients; (2) to minimize the risk of overfitting, the least absolute shrinkage and selection operator (LASSO) Cox regression was applied, and the penalty parameter was estimated by tenfold cross-validation in the training set at the minimum partial likelihood deviance; (3) multivariate Cox regression analysis was ultimately performed to screen the optimal m6A-related lncRNAs for the prognostic signature. Then, a prognostic signature for the patients was developed using multivariate regression coefficients of lncRNA expression. The risk score was calculated by the formula as follows:${\text{Risk}}\;{\text{score}} = \beta 1 * {\text{Exp}}1 + \beta 2 * {\text{Exp}}2 + \beta 3 * {\text{Exp}}3,$ where β is the coefficient and Exp is the expression value of the corresponding m6A-related lncRNAs. According to the median of risk scores as a cut-off value, patients were divided into high-risk and low-risk groups. Kaplan–Meier analysis was used to show the survival difference between the high- and low-risk groups. The receiver operating characteristic (ROC) curves were conducted for assessment of the predictive ability of the signature by “SurvivalROC” R package.

To distinguish patients from high- to low-risk group, the optimal cutoff point for the risk score was determined using the formula described above in the training set. We performed Kaplan–Meier analysis to show the survival difference between the high- and low-risk groups. The predictive ability of the prognostic signature was assessed by the time-dependent ROC curve.

### Immune infiltration analysis

To investigate the immune infiltration of PDAC, we analyzed the infiltrating score of 16 immune cells and 13 immune-related pathways from PDAC gene expression profiles using single-sample gene set enrichment analysis (ssGSEA)^19^. We further used “estimate” R package to calculate the immune, stromal, and ESTIMATE scores of each PDAC patient based on the ESTIMATE algorithm^20^. Finally, we extracted potential immune checkpoints from previous literature, then compared and analyzed differences among them at *P*-value < 0.05.

### Mutation and drug sensitivity analyses

The waterfall function of “maftools” R package was used to visualize the mutation landscape in patients with high- and low-risk group. The somatic mutation count and TMB (mutations per million bases) of each patient were calculated. Wilcoxon test was performed to compare the somatic mutation and TMB levels between the high- and low-risk group. To explore differences in therapeutic effects of chemotherapeutic drugs in patients across the high- and low-risk groups, R package “pRRophetic” was used to predict the half-maximal inhibitory concentration (IC50), which could construct a ridge regression signature based on TCGA gene expression profiles and Genomics of Drug Sensitivity in Cancer cell line expression spectrum^21^.

### Clinical correlation analysis and stratification analysis of signature

Clinical correlation analysis was conducted to evaluate the correlation between risk score of the prognostic signature and clinical factors, including age, gender, T and N stage, surgery type, tumor size, and tumor location. In addition, we also divided the clinical characteristics of patients into various subgroups, namely those < 60 and > 60 years old, males and females, those with high and low pathological grades, T1-2 and T3-4, N0 and N1, body/tail of pancreas and head of pancreas, Whipple and other surgery types, as well as tumor size < 3.0 cm and > 3.0 cm. Thereafter, we calculated risk scores and compared them across subgroups. In addition, we used the risk scores to divide each sub-group into low- and high-risk groups, then generated K-M curves for comparison.

### Development and validation of prognostic nomogram

We used Cox regression analysis to select clinical prognostic factors along with risk status as the prognostic parameters to construct a nomogram model for predicting the probability of survival at 1 and 3 years in PDAC patients and then plotted the nomogram by the “rms” R packages. Calibration plots were used to evaluate the discriminative power of the nomogram. The ROC curve and calibration curve varying with time were also drawn to estimate the accuracy of the actual observed rate with the predicted survival for 1- and 3-year OS of the nomogram.

### Functional annotation of the included m6A-related lncRNAs

To evaluate the possible biological functions of the included m6A-related lncRNAs, Gene set enrichment analysis (GSEA) was performed to assess whether predefined gene sets showed statistically significant differences based on the risk score. “Expression datasets” and “phenotype labels” were made and imported as required by the software, and the “gene set database” was selected “C2cp.kegg.v7.2.symbols.gmt”, the number of permutations was set to 1000 times, and the “phenotype labs” were set to high-risk score versus low-risk score. The outcomes meet *P*-value < 0.05 were included in the analysis, and FDR < 0.25 were considered as the significant difference criteria.

### Cell lines and transfection

The normal pancreatic cell line hTERT-HPNE and two human PDAC cell lines (PANC-1, SW1990) were purchased from the American Type Culture Collection (ATCC) and the Type Culture Collection of the Chinese Academy of Sciences (Shanghai, China). We cultured in RPMI 1640 (Thermo Fisher, Waltham, MA, USA) supplemented with 10% FBS (Thermo Fisher). hTERT-HPNE cells were maintained in DMEM medium supplemented with 5% FBS, human epidermal growth factor (EGF 10 ng/mL, ThermoFischer, Waltham, MA, USA), puromycin (750 ng/mL, ThermoFischer, Waltham, MA, USA) and 5 mM D-glucose. All cells were cultured in a humidified incubator containing 5% CO2 at 37 °C. Small interfering RNAs (siRNAs) targeting lncRNA DCST1-AS1 and negative control siRNA (si-NC) were synthesized by GenePharma. (Shanghai, China). Transfection was carried out using Lipofectamine 2000 (Invitrogen, Carlsbad, CA, USA) and the final concentration was 100 nm for siRNAs.

### RNA extraction and qRT-PCR analysis

RNA extraction from tissues was performed using TRIzol reagent (Invitrogen, Carlsbad, CA, USA). The total RNA was transcribed to cDNA using Prime ScriptTM RT Master Mix (Takara, Japan). Real-time qPCR analyses were quantified with Bio-Rad system, and expression levels were normalized to GAPDH levels.

### Cell counting Kit-8 (CCK-8) assay

To test cell viability, CCK-8 assay reagent (Beyotime, Shanghai, China) was utilized. SW1990 cells were inoculated in 96-well plates and then added with CCK-8 reagent at indicated time points. After the cells were cultured for another 4 h, the absorption at 450 nm was recorded.

### Wound healing and transwell assays

Cell migration was assessed by wound healing assay. Briefly, cells were seeded into 6-well plates and allowed to grow until confluent. Following serum starvation for 24 h, an artificial wound was created onto the cell monolayer using a sterile 100 μl tip. Then, the cells were washed with serum-free medium, added with complete medium, and images of the wounds were collected at 0 h and 24 h.

Transwell chambers (24-well insert, 8 μm, Corning Costar Corp, USA) were performed to evaluate the migration capability of pancreatic cancer cells. Briefly, transfected cells in serum-free medium were added into the top chamber and the complete medium was supplemented into the bottom medium. After 24 h of incubation, the migrated cells were dyed using crystal violet (Beyotime). The dyed cells were counted in three fields under a 200 × objective lens using a microscope.

## Results

### Identification of m6A-related prognostic lncRNAs in PDAC patients

Supplementary Fig. 1 shows the flowchart of data analysis in this study. The detailed clinicopathological features of PDAC patients are summarized in Supplementary Table 1. Through Pearson correlation analysis, we obtained 317 m6A-related lncRNAs (correlation coefficient > 0.4, *P*-value < 0.001; Supplementary Fig. 2). Subsequently, we identified 95 lncRNAs related to OS (*P*-value < 0.05) using univariate Cox regression analysis (Supplementary Table 2).

### Establishment and validation of m6A-related prognostic lncRNA signature

**Figure 1:**
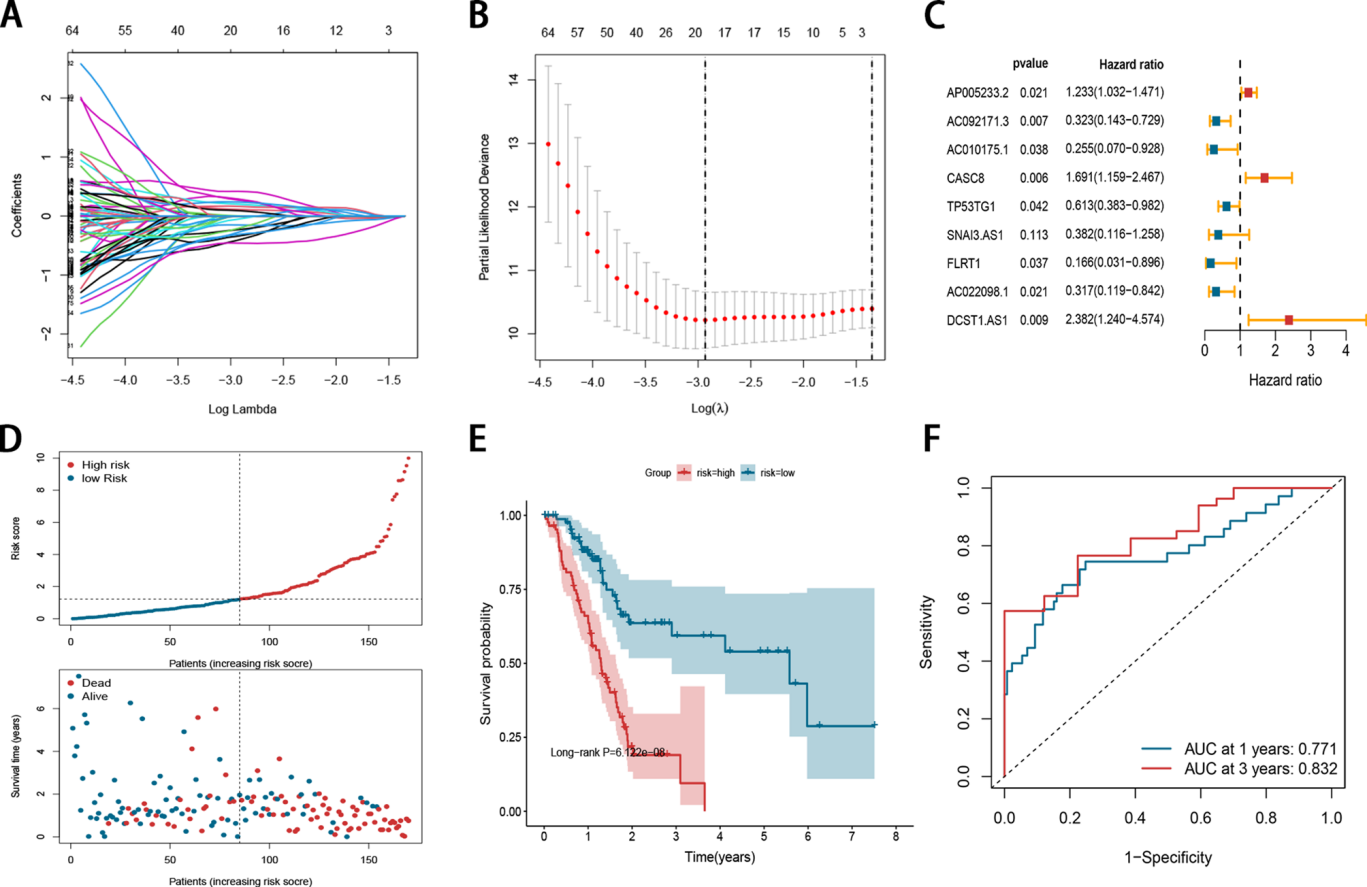
Construction of m6A-related lncRNA signature in PDAC patients. (**A**, **B**) The LASSO regression analysis and partial likelihood deviance of the 19 m6A-related lncRNAs. (**C**) Forrest plot showed that a total of 9 m6A-related lncRNAs were identified as prognosis related by multivariate cox analysis. (**D**) The distribution of the risk scores and patient living status. (**E**) Kaplan–Meier survival estimates of OS according to the signature. (**F**)The 1- and 3-year ROC curves of the signature in predicting OS of PDAC patients.

Based on 95 candidate prognostic lncRNAs, LASSO regression analysis was performed, 19 m6A-related lncRNAs remained according to the minimum partial likelihood deviance (Fig. 1A, B). Then an optimum prognostic signature involving 9 m6A-related lncRNAs was ultimately defined based on multivariate Cox regression analysis (Fig. 1C). The risk score for each patient was calculated as follows: risk score = (0.20906 × Exp AP005233.2) + (−1.13087 × ExpAC092171.3) + (−1.36500 × ExpAC010175.1) + (0.52510 × ExpCASC8) + (−0.48871 × ExpTP53TG1) + (−0.96255 × ExpSNAI3.AS1) + (−1.79504 × ExpFLRT1) + (−1.14898 × ExpAC022098.1) + (0.86774 × ExpDCST1.AS1). Among 9 lncRNAs, 3 lncRNAs were regarded as risk factors, while other 6 lncRNAs were protective factors. The distribution of the risk scores, OS statuses, and OS times of PDAC patients were visualized by scatter plots (Fig. 1D). Patients were divided into high-risk group and low-risk group based on medium value of the risk score, and the low-risk group have a lower mortality than high-risk group (Fig. 1E). For assessing the prediction performance of the prognostic signature, a risk score was calculated for each patient. The ROC curves demonstrated that the signature harbored a promising performance to predict OS of PDAC in the TCGA cohort (1-year AUC = 0.771, 3-year AUC = 0.832; Fig. 1F).

**Figure 2:**
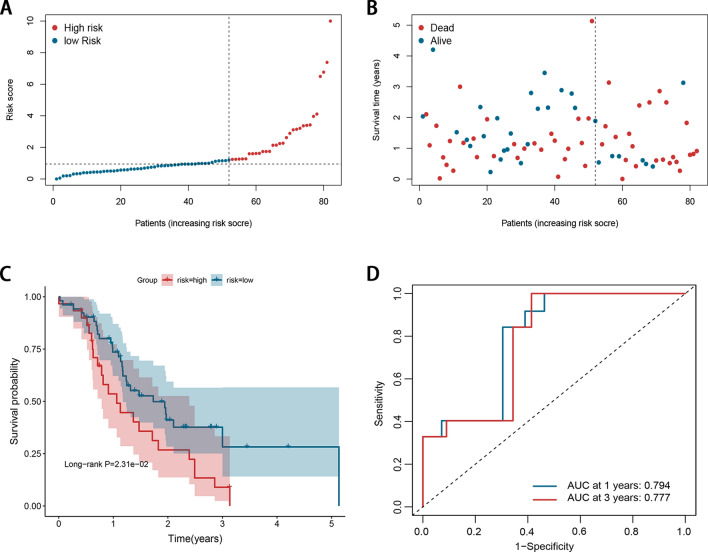
Evaluation of the signature in the ICGC dataset. (**A**) The distribution of the risk scores. (**B**) Scatter plot illustrating the patients’ survival status. (**C**) Kaplan–Meier survival estimates of OS according to the signature. (**D**) The 1- and 3-year ROC curves of the signature in predicting OS of PDAC patients.

We used the ICGC dataset to determine the prognostic accuracy of the signature (Fig. 2). We calculated the risk score of the prognostic signature for each patient in the validation dataset using the same formula. The distribution of the risk scores, OS statuses, and OS times of PDAC patients were visualized by scatter plots (Fig. 2A, B). Kaplan–Meier survival analysis was conducted and demonstrated that the high-risk group exhibited a significantly poorer prognosis compared with the low-risk group (Fig. 2C). These figures indicated that high-risk patients were corresponded to more death cases and shorter OS time compared with low-risk patients. The AUC of ROC for 1- and 3-year survival predictions were 0.794 and 0.777 in the ICGC dataset (Fig. 2D).

### Immune infiltration analysis

**Figure 3:**
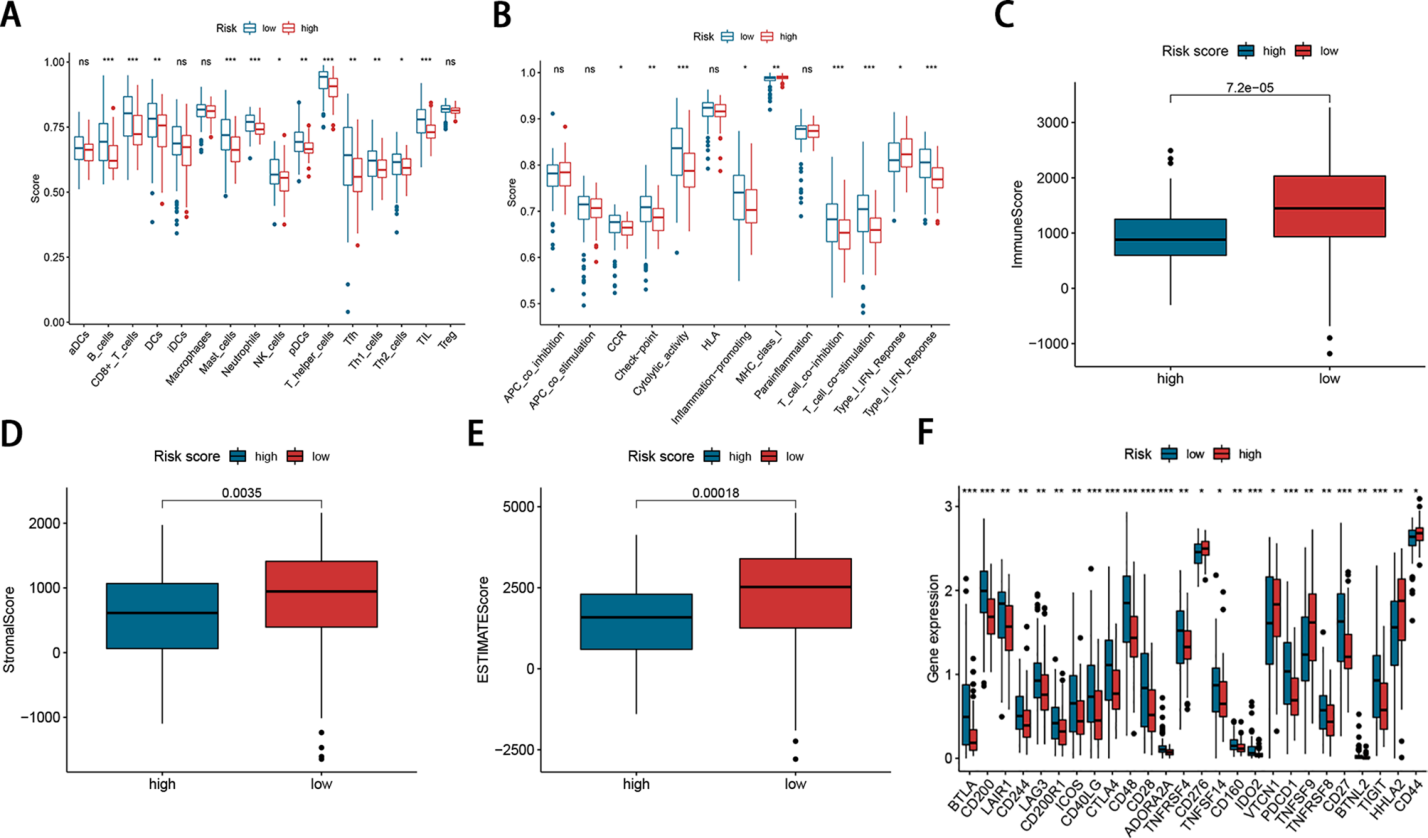
Immune infiltration analysis between the two risk groups. (**A**, **B**) Boxplot of immune cells and immune function between high and low risk groups. (**C**–**E**) Association the risk score and the TME score (immune scores, stromal scores, and ESTIMATE scores). (**F**) Expression of immune checkpoints in high and low risk groups.

To further explore the relationship between the prognostic risk score and tumour-infiltrating immune-cell fractions, we quantified the enrichment scores of diverse immune cell subpopulations. As shown in Fig. 3A, the high-risk score was negatively associated with lower abundance of immune infiltrating cells like B cells, CD8 + T cells, DCs, mast cells, neutrophils, NK cells, pDCs, T helper cells, Tfh, Th1 cells, Th2 cells, and TIL cells (*P*-value < 0.05). In addition, significant differences between two risk groups were observed in 9 of 13 immune-related pathways (*P*-value < 0.05; Fig. 3B). Analysis of relationship between the risk score and the TME score revealed that the high-risk score was significantly associated with low stromal score, immune score, and ESTIMATE score (Fig. 3C–E). In addition, as the immune checkpoint inhibitors (ICIs) are currently considered to be a potential curative approach, and a mass of ICIs are under clinical trial. Here, we analyzed the relationship between the immune checkpoints and our risk score. The result demonstrated that 26 immune checkpoints were differentially expressed in two groups, including PDCD1 (PD-1), CTLA4 (Fig. 3F), suggesting a potential role of the signature model in predicting immune responses to immunotherapy in PDAC patients.

### Somatic mutation landscapes and drug sensitivity analyses

**Figure 4:**
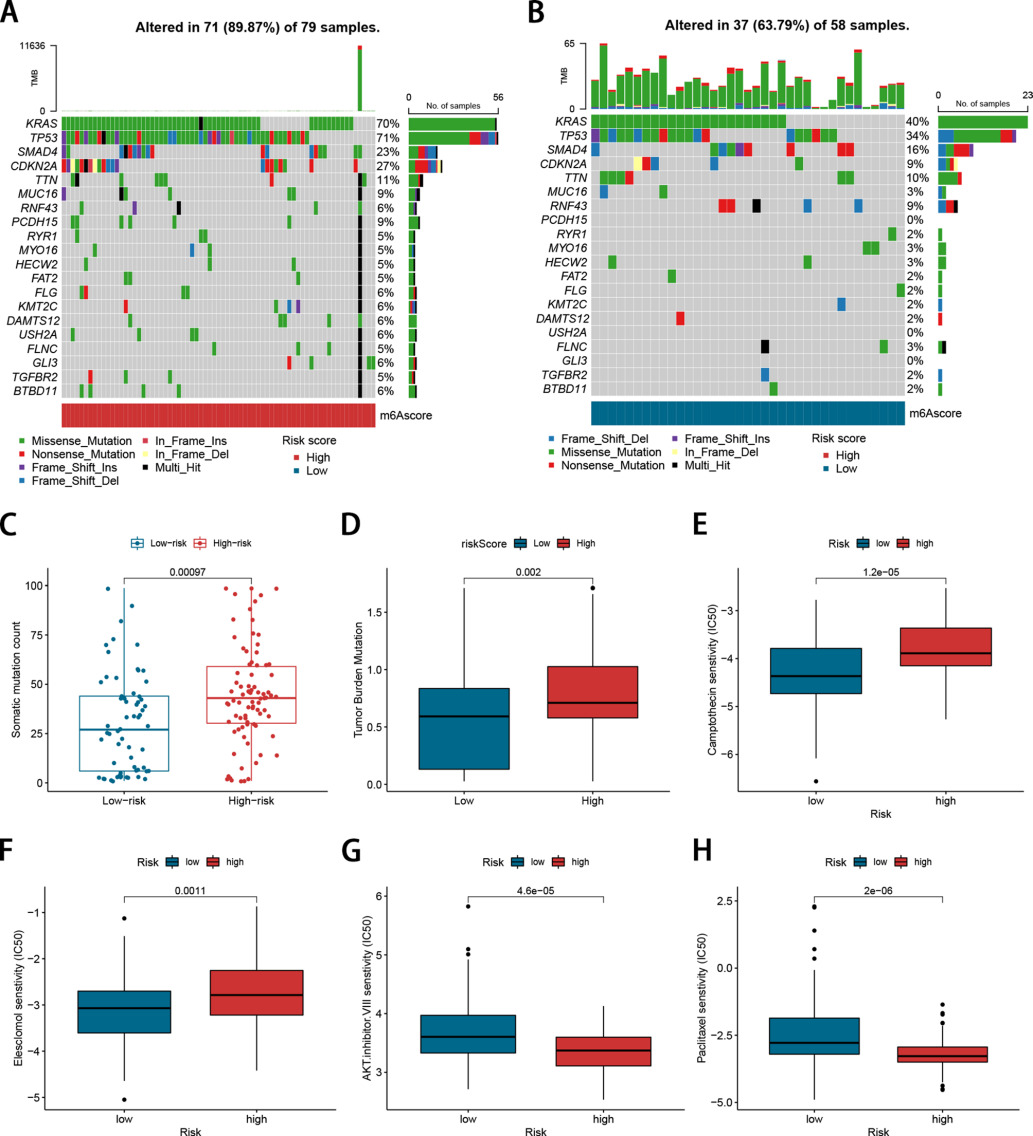
Comparison of the somatic mutations and drug sensitivity analyses between the two risk groups. (**A**, **B**) The waterfall plot of somatic mutation features established with the high-risk group and the low-risk group. Each column represented an individual patient. The upper barplot showed TMB, the number on the right indicated the mutation frequency in each gene. The right barplot showed the proportion of each variant type. (**C**) Correlation of somatic mutation count with risk score. (**D**) Correlation of TMB with risk score. (**E**–**H**) The IC50 values of four chemotherapeutic drugs in the high- and low-risk groups.

We explored the differences in somatic mutation between high- and low-risk groups based on the TCGA cohort (Fig. 4A, B). Waterfall plots depicted the frequently mutated genes in PDAC stratified by high- and low-risk groups. The top 5 mutated genes were KRAS, TP53, SMAD4, CDKN2A, TTN. We noticed that the high-risk group exhibited more frequent somatic mutations than the low-risk group. Next, we assessed the relationship of the signature with the somatic mutation count and tumor mutation burden (TMB). The results indicated that the somatic mutation count (*P*-value < 0.001; Fig. 4C) and TMB (*P*-value = 0.002; Fig. 4D) of patients in the high-risk group were significantly higher than in the low-risk group. We used the R package “pRRophetic” to estimate the response to chemotherapy drugs in the high- and low-risk groups. As shown in Fig. 4E–H, patients in the low risk group were more sensitive to Camptothecin and Elesclomol, while those in the high risk group were more sensitive to AKT.inhibitor.VIII and Paclitaxel. However, there is no statistical difference in the response to cisplatin and gemcitabine between the two risk groups (Supplementary Fig. 3).

### Clinical correlation analysis and stratification analysis of signature

**Figure 5:**
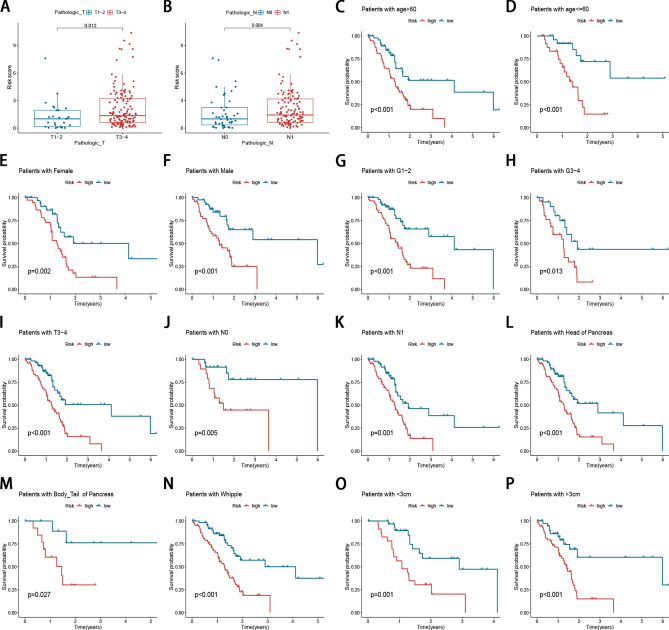
Clinical correlation analysis and stratification analysis of signature. (**A**) Correlation between the risk score and T stage. (**B**) Correlation between the risk score and N stage. (**C**–**P**) Stratified survival analysis between the high- and low-risk group.

To further verify the significance of the prognostic signature in clinical practices, we examined the correlation between the prognostic signature and the available clinical characteristics. As shown in Fig. 5, there were significant differences between the high- and low-risk groups regarding T stage (*P* = 0.012; Fig. 5A) and N stage (*P* = 0.004; Fig. 5B). To confirm the prognostic discriminatory power of the signature, we performed stratified survival analysis in various clinical subgroups, including age (age < 60 and age > 60), gender (female and male), grade (G1-2 and G3-4), T stage (T1-2 and T3-4), N stage (N0 and N1), tumor location (body/tail and head), surgery type (Whipple and other), and tumor size (< 3.0 cm and > 3.0 cm). As the result shown in Fig. 5C–P, the OS of the high-risk patients based on age (*P*-value < 0.001), sex (*P*-value = 0.002 in female and *P*-value < 0.001 in male), grade (*P*-value < 0.001 in G1-2 and *P*-value = 0.013 in G3-4), T stage (*P*-value < 0.001 in T3-4), N stage (*P*-value = 0.005 in N0 and *P*-value = 0.001 in N1), tumor location (*P*-value < 0.001 in head and *P*-value = 0.027 in body/tail), surgery type (*P*-value < 0.01 in Whipple), tumor size (*P*-value = 0.001 in < 3.0 cm and *P*-value < 0.001 in > 3.0 cm) were significantly lower than those of the low-risk patients.

### Construction of prognostic nomogram

**Figure 6:**
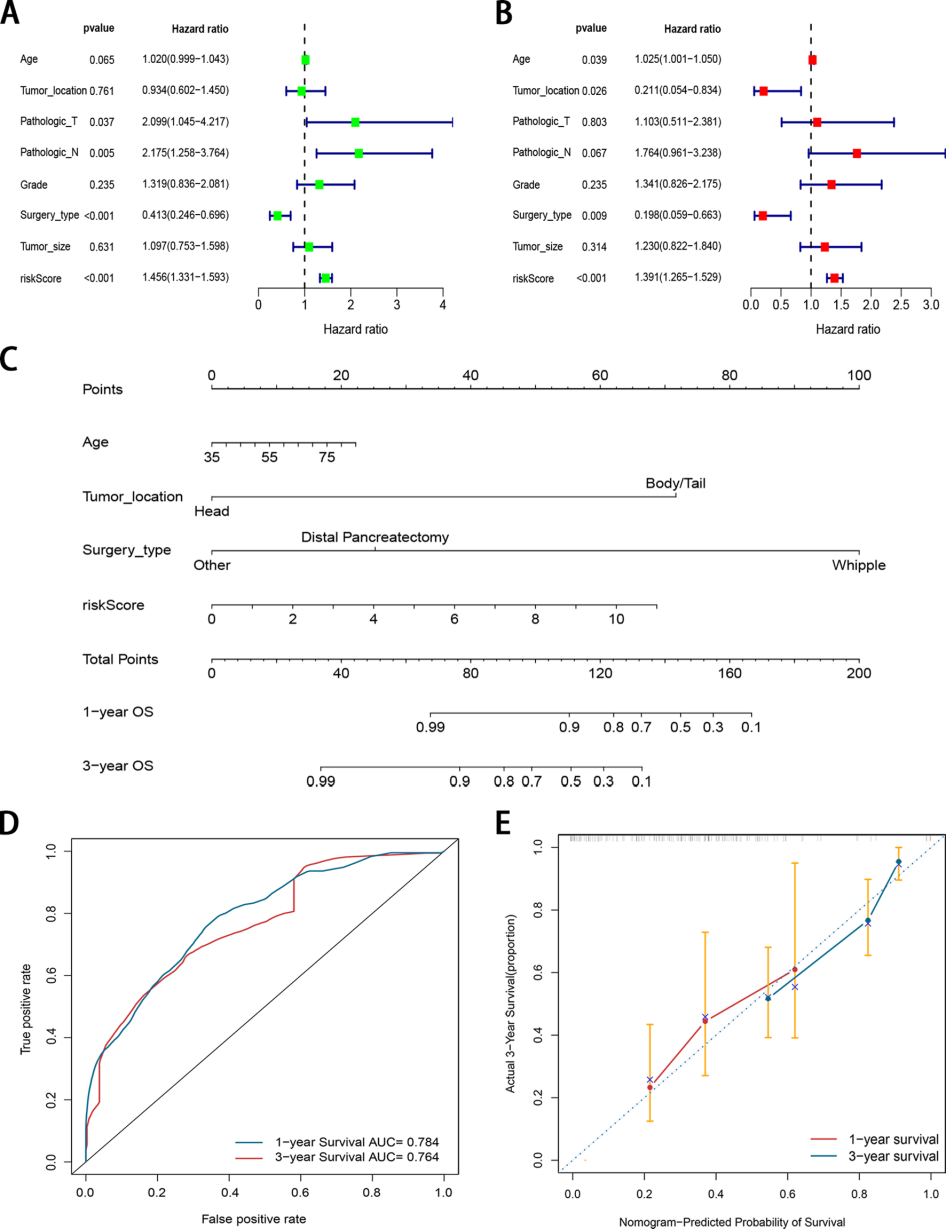
Development of a nomogram for survival prediction of PDAC patients based on signature and clinical characteristics. (**A**, **B**) Univariate and multivariate Cox analyses simultaneously demonstrated the independent prognostic value of the risk score. (**C**) The nomogram combining risk signature and clinicopathological factors. (**D**) AUCs on the nomogram suggested that this model had higher accuracy in OS at 1 and 3 years. (**E**) Calibration plots were established to compare the proposed nomogram with an ideal model.

Univariate and multivariate Cox regression analyses were employed to determine whether the m6A-related lncRNA signature has prognostic value independent of clinicopathological indicators, such as age, tumor location, T and N stage, grade, surgery type, and tumor size. In addition, to determine whether there is collinearity among the potential risk factors, we performed a correlation analysis among the potential risk factors. The result demonstrated that there was no obvious correlation between them (Table S3). As shown in Fig. 6A, B, univariate and multivariate analyses revealed a significant correlation between OS of PDAC patients, age, tumor location, surgery type, and risk score. Nomogram for the 1- and 3-year OS rates based on the independent predictors determined from the multivariate analysis is shown in Fig. 6C. A certain point was generated for each covariate, and a total nomogram score, which was correlated with the 1- and 3-year OS rates, was calculated for every patient. The AUCs for the 1- and 3-year OS predictions were 0.784 and 0.764, respectively (Fig. 6D). Calibration curves were drawn to depict the predictive value between the predicted 1-and 3-year survival events and the virtual observed outcomes (Fig. 6E), which showed that the nomogram was precise and stable.

### Gene set enrichment analysis (GSEA)

Gene set enrichment analyses revealed functions and pathways in the high-risk group that were mainly enriched in cell cycle, P53 singaling pathway, mismatch repair, and DNA replication signaling pathways (Supplementary Fig. 4). Notably, most of these functions were closely associated with occurrence and development of tumors.

### Silencing of DCST1-AS1 suppresses the proliferation and migration of PDAC cells in vitro

**Figure 7:**
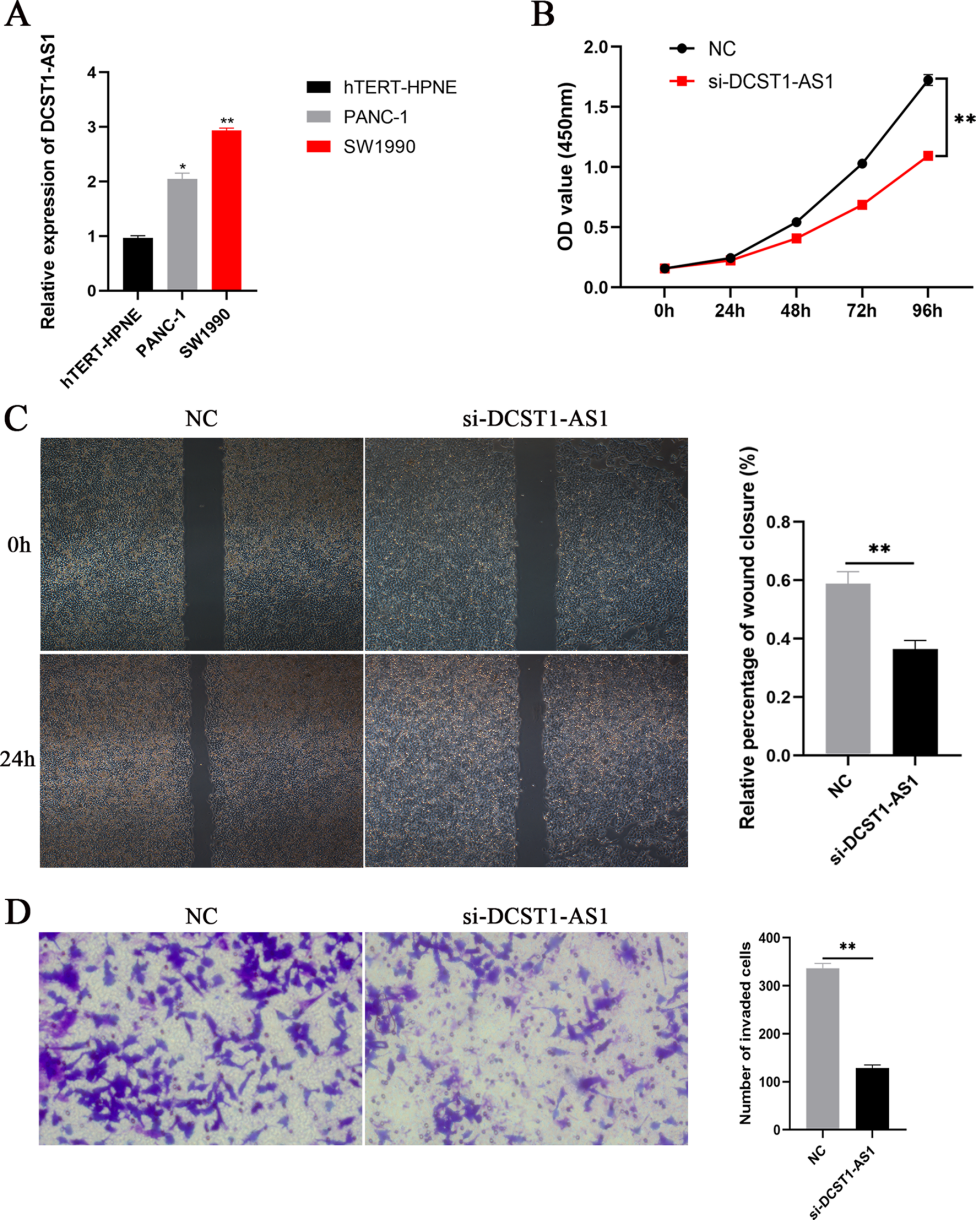
Impacts of DCST1-AS1 knockdown on the proliferation and migration of PDAC cells in vitro. (**A**) The expression of DCST1-AS1 in PANC-1 and SW1990 cells was detected by qRT-PCR assay. (**B**) The viability of SW1990 cells was analyzed by CCK-8 assay. (**C**, **D**) The migration capability of SW1990 cells transfected with siRNA were measured using wound healing assay and transwell assay.

Combined with the results of the database, the functional phenotypic role of DCST1-AS1 was investigated by experimental studies. To verify the expression of DCST1-AS1 in PDAC cells, we firstly performed qRT-PCR assay. As shown in Fig. 7A, DCST1-AS1 was significantly upregulated in PDAC cells (PANC-1 and SW1990) compared with that in the normal pancreatic cell line hTERT-HPNE (*P*-value < 0.05). Then, siRNA specific for DCST1-AS1 was used to downregulate the expression of DCST1-AS1 in SW1990 cells. CCK-8 assay indicated that DCST1-AS1 knockdown repressed the viability of SW1990 cells relative to the negative control (Fig. 7B).

Given that PDAC is more prone to metastasize, we further studied whether DCST1-AS1 affected the invasion of PDAC cells. As a result of the wound healing assay, knockdown of DCST1-AS1 significantly decreased the migration rate of SW1990 cells (Fig. 7C). Consistently, transwell invasion assays confirmed that the migration ability was significantly inhibited after knockdown of DCST1-AS1 in DCST1-AS1 cells (Fig. 7D). All these findings suggested that DCST1-AS1 knockdown inhibited PDAC cell progression.

## Discussion

PDAC is a complex and heterogeneous tumor that is associated with a high morbidity and poor prognosis^22^. Current major challenges of PDAC are the early diagnosis, precise prediction of tumor progression and effective intervention. Thus, it is urgent to identify specific biomarkers that can improve accurate prognosis prediction that can guide personalize treatment selection to improve survival rates. With the development of next-generation sequencing technology, genome sequencing and analysis can be performed within a clinically significant turnaround time. This can determine the treatment goals of individual patients and personalize treatment selection. Incorporating preclinical findings and molecular-guided therapies into the design of clinical trials has the potential to significantly improve the outcome of this deadly malignant tumor^23^. The m6A regulators reportedly act as a lncRNA structural switch, participates in the lncRNA-mediated competing endogenous RNA model, and enhances the stability of lncRNA to serve its functions, thereby influencing tumor initiation and progression^5,6,24^. Therefore, it is necessary to further study m6A-related lncRNAs to clarify the potential regulatory mechanism of m6A-related lncRNAs in tumor immune microenvironment.

Taking advantages of high-throughput RNA-seq data and aided by previous publications for experimentally supported lncRNAs related to m6A modification, we constructed and verified a risk stratification signature to improve the prediction of clinical outcome of PDAC. First, we identified 95 lncRNAs related to OS through Pearson correlation and univariate Cox regression analyses. Next, a robust m6A-related lncRNA signature was established with the combination of LASSO and multivariate Cox regression analyses. Patients with high-risk score had a worse OS compared with low-risk score patients. The favorable performance of this signature was confirmed by ROC curve, and an independent ICGC dataset. Furthermore, stratified survival analysis in various clinical subgroups confirmed the robust prognostic discriminatory power of the signature. Analysis of immunocyte infiltration by signature as well as immunologic function, revealed significant differences in two risk score groups. Similarly, analyses of TME score, immune checkpoints, and drug susceptibility also revealed significant differences between low- and high-risk groups. Further analysis revealed that patients with high-risk had significantly higher more frequent somatic mutations and TMB than their low-risk counterparts, indicating that those in the high-risk group had a close relationship with genomic instability. Results from univariate and multivariate cox analyses revealed that the prognostic value of signature was independent of other clinical characteristics. To improve the usefulness of the prognostic signature, we established a nomogram model, including age, tumor location, surgery type, and risk score. This model had excellent accuracy in predicting 1- and 3-year survival rates of patients with PDAC. Finally, the functional phenotypic role of DCST1-AS1 was investigated by experimental studies. We validated the expression of DCST1-AS1 in different PDAC cells and normal pancreatic cell lines. In vitro analysis showed that inhibition of DCST1-AS1 suppresses the proliferation and migration of PDAC cells.

A growing number of studies have highlighted the potential effects of the cancer immune microenvironment on the development and progression of cancer^25^. Assessment of the enrichment of tumour infiltrates may help to predict the prognosis and host immune response to tumour antigens of cancer patients. In the present study, we found that compared to the high-risk group, the low-risk group had a higher proportion of B cell. Accumulating evidence have shown that B cells also participated in immune response^26,27^. The enrichment of B cells and tertiary lymphoid structures (TLS) have been considered as the strongest prognostic factor of prolonged survival and positively correlated with the response to PD1 blockade in soft-tissue sarcomas^28^. Meanwhile, B cell-related genes were markedly higher expressed in patients who responded to immune checkpoint blockade compared to non-responders^27^. T cells has been deemed to play a vital role in anti-cancer immune response. This is corresponded to our result that low-risk group patients contained more CD8 + T cell than low-risk group in TME. This provides evidence that the prognostic signature might predict the efficacy of immunotherapy. Advances in the knowledge of immune checkpoint inhibitors have uncovered a new era of cancer immunotherapy^29^. However, only a limited proportion of treated patients possess clinical responses, indicating the urgent demands for the investigation of predictive models. Therefore, we performed an immune checkpoint analysis and found 26 immune checkpoints with significant difference. Amon them, PD1 and CTLA4 were well-known predictive biomarkers for immunotherapy response^30^. In addition to PD-1 and CTLA-4, our risk model revealed some underlying checkpoints that might be novel targets for immunotherapy in PDAC, especially for patients who are non-responders of anti-PD1/PDL1 treatment, given the fact that not all patients are beneficial from anti-PD1/PDL1 strategy.

PDAC was found with widespread and complex patterns of chromosomal rearrangement, which means it was necessary^31^. We compared the somatic mutation data of high-risk group and low-risk group, and found that the mutation frequency of *TP53* in the high-risk group was significantly increased (71% versus 34%). Considering the fact that *TP53* is a recognized tumor suppressor gene, this might also explain the better prognosis in low-risk group. In addition, *KRAS* mutation is the main event of PDAC; it confers permanent activation of the KRAS protein to maintain the cellular processes of proliferation, transformation, invasion and survival^32^. Patients with *KRAS* mutation were associated with poor prognosis of PDAC. Therefore, it is meaningful to further explore potential ability of *KRAS* mutation to predict prognosis in PDAC. Moreover, our results indicated that TMB was significantly higher in the high-risk group, from the perspective that TMB was widely accepted as a biomarker for predicting immunotherapy^33^, we speculated that the high-risk patients might get more benefits from immunotherapy.

Although most lncRNAs in our signature have not been reported before, some of them have been proved to be associated with cancer development, including PDAC. A recent study revealed that lncRNA DCST1-AS1 expression was significantly upregulated in triple-negative breast cancer (TNBC), and promote TGF-β-induced epithelial-mesenchymal transition and enhance chemoresistance in TNBC cells through ANXA1^34^. Similarly, the expression of TP53TG1 was markedly elevated in PDAC and knockdown of GPX4 in vitro and in vivo inhibited cell proliferation, promoted apoptosis, and decreased migration and invasion by miR-96/KRAS axis^35^. CASC8 was found highly expressed in retinoblastoma, contributing to retinoblastoma cell proliferation by upregulating miR34a methylation^36^. Zhang and colleagues^37^ recently revealed that lncRNA SNAI3-AS1 promoted PEG10-mediated proliferation and metastasis of hepatocellular carcinoma cell via decoying of miR-34a-5p and miR-27a-3p.

In this work, altogether 4 variables were identified to independently predict prognosis, which were age, tumor location, surgery, and risk score. Among them, age is identified as the vital factor that affects OS in some articles^38,39^. The location of the PDAC is critical because it determines the extent of resection. In addition to guiding surgical selection, tumor location can actually predict survival in patients with PDAC^38,40–42^. The use of tumor location to prognosticate PDAC is appealing because this information can be easily obtained by cross-sectional imaging. Atsushi et al.^38^ built a nomogram based on data from the National Cancer Database, and identified 8 variables. They showed by multivariate analysis that tumor location is an independent prognostic factor, consistent with our results. Winer et al.^40^ analyzed 175,556 patients with PDAC undergoing curative resection and found that patients with tumor of the head had worse OS compared with those with tumors of the body and tail. A single-center observational study showed that patients with head PDAC had worse OS and disease-free survival (DFS) compared with those with body and tail tumors^41^. Similarly, significant differences of the median disease-specific survival (DSS) between patients with head and those with body/tail (26 months vs 33 months)^42^.

However, we have to admit that some limitations were also existing in our study. Firstly, although we constructed our proposed signature using biostatistical methods and validated it using a dataset from the ICGC database, we did not validate it experimentally or clinically. Secondly, most patients were diagnosed as stage III, which may be the main reason responsible for tumor stage was not an independent prognostic factor. Thirdly, it involves the nature of retrospective study. Intratumor heterogeneity contributes to sampling bias, which means a small tissue sample may not be representative of the whole tumor mass. Finally, experimentally supported m6A-related lncRNAs are extremely limited in number. There will be an increasing number of experimentally confirmed cancer metastasis-related lncRNAs in the future.

## Conclusions

The m6A-related lncRNA signature may act as a novel independent prognostic biomarker for screening patients who may have a probability to benefit from the individualized treatment. Our proposed signature could not only be a useful tool for prognostic evaluation, but also be complementary with and add information to the predictive biomarkers of immunotherapy response in patients with PDAC.

## Supplementary Information

Supplementary Information 1.

Supplementary Information 2.

## Data Availability

Publicly available datasets were analyzed in this study. This data can be found here: TCGA database (http://www.cancer.gov/tcga) and ICGC database (https://icgc.org/).

## Abbreviations

PDAC: Pancreatic ductal adenocarcinoma
m6A: N6-methyladenosine
lncRNAs: Long non-coding RNAs
LASSO: Least absolute shrinkage and selection operator
ROC: Receiver operating characteristic
TME: Tumor microenvironment
RNA-seq: RNA-sequencing
TCGA: The cancer genome atlas
ICGC: International cancer genome consortium
FPKM: Fragments perkilobase million
GSEA: Gene set enrichment analysis
ICIs: Immune checkpoint inhibitors

## References

[1] Mizrahi JD, Surana R, Valle JW, Shroff RT (2020). Pancreatic cancer. Lancet.

[2] Rahib L (2014). Projecting cancer incidence and deaths to 2030: the unexpected burden of thyroid, liver, and pancreas cancers in the United States. Cancer Res..

[3] Rombouts SJ (2015). Systematic review of innovative ablative therapies for the treatment of locally advanced pancreatic cancer. Br. J. Surg..

[4] Gbolahan OB, Tong Y, Sehdev A, O'Neil B, Shahda S (2019). Overall survival of patients with recurrent pancreatic cancer treated with systemic therapy: a retrospective study. BMC Cancer.

[5] Du K, Zhang L, Lee T, Sun T (2019). m(6)A RNA methylation controls neural development and is involved in human diseases. Mol. Neurobiol..

[6] Wang S (2017). Roles of RNA methylation by means of N(6)-methyladenosine (m(6)A) in human cancers. Cancer Lett..

[7] He L (2019). Functions of N6-methyladenosine and its role in cancer. Mol. Cancer.

[8] Ma S (2019). The interplay between m6A RNA methylation and noncoding RNA in cancer. J. Hematol. Oncol..

[9] Li T (2019). METTL3 facilitates tumor progression via an m(6)A-IGF2BP2-dependent mechanism in colorectal carcinoma. Mol. Cancer.

[10] Wang Q (2020). METTL3-mediated m(6)A modification of HDGF mRNA promotes gastric cancer progression and has prognostic significance. Gut.

[11] Zhang J (2019). Excessive miR-25-3p maturation via N(6)-methyladenosine stimulated by cigarette smoke promotes pancreatic cancer progression. Nat. Commun..

[12] Schmitt AM, Chang HY (2016). Long noncoding RNAs in cancer pathways. Cancer Cell.

[13] Xu S (2020). Long noncoding RNAs control the modulation of immune checkpoint molecules in cancer. Cancer Immunol. Res..

[14] Statello L, Guo CJ, Chen LL, Huarte M (2021). Gene regulation by long non-coding RNAs and its biological functions. Nat. Rev. Mol. Cell Biol..

[15] Chen Y, Lin Y, Shu Y, He J, Gao W (2020). Interaction between N(6)-methyladenosine (m(6)A) modification and noncoding RNAs in cancer. Mol. Cancer.

[16] He Y (2018). ALKBH5 inhibits pancreatic cancer motility by decreasing long non-coding RNA KCNK15-AS1 methylation. Cell. Physiol. Biochem..

[17] Hu X (2020). IGF2BP2 regulates DANCR by serving as an N6-methyladenosine reader. Cell Death Differ..

[18] Chen JQ (2021). M(6)A-mediated up-regulation of LncRNA LIFR-AS1 enhances the progression of pancreatic cancer via miRNA-150-5p/VEGFA/Akt signaling. Cell Cycle.

[19] Rooney MS, Shukla SA, Wu CJ, Getz G, Hacohen N (2015). Molecular and genetic properties of tumors associated with local immune cytolytic activity. Cell.

[20] Yoshihara K (2013). Inferring tumour purity and stromal and immune cell admixture from expression data. Nat. Commun..

[21] Geeleher P, Cox NJ, Huang RS (2014). Clinical drug response can be predicted using baseline gene expression levels and in vitro drug sensitivity in cell lines. Genome Biol..

[22] Chen H, Zhuo Q, Ye Z, Xu X, Ji S (2021). Organoid model: a new hope for pancreatic cancer treatment?. Biochim. Biophys. Acta Rev. Cancer.

[23] Dreyer, S. B., Chang, D. K., Bailey, P. & Biankin, A. V. Pancreatic cancer genomes: implications for clinical management and therapeutic development. *Clin. Cancer Res. Off. J. Am. Assoc. Cancer Res.* **23**, 1638–1646, doi:10.1158/1078-0432.Ccr-16-2411 (2017). 10.1158/1078-0432.CCR-16-2411 28373362

[24] Ban Y (2020). LNCAROD is stabilized by m6A methylation and promotes cancer progression via forming a ternary complex with HSPA1A and YBX1 in head and neck squamous cell carcinoma. Mol. Oncol..

[25] Seager RJ, Hajal C, Spill F, Kamm RD, Zaman MH (2017). Dynamic interplay between tumour, stroma and immune system can drive or prevent tumour progression. Conver. Sci. Phys. Oncol..

[26] Cabrita R (2020). Tertiary lymphoid structures improve immunotherapy and survival in melanoma. Nature.

[27] Helmink BA (2020). B cells and tertiary lymphoid structures promote immunotherapy response. Nature.

[28] Petitprez F (2020). B cells are associated with survival and immunotherapy response in sarcoma. Nature.

[29] Darvin P, Toor SM, Sasidharan Nair V, Elkord E (2018). Immune checkpoint inhibitors: recent progress and potential biomarkers. Exp. Mol. Med..

[30] Havel JJ, Chowell D, Chan TA (2019). The evolving landscape of biomarkers for checkpoint inhibitor immunotherapy. Nat. Rev. Cancer.

[31] Waddell N (2015). Whole genomes redefine the mutational landscape of pancreatic cancer. Nature.

[32] Buscail L, Bournet B, Cordelier P (2020). Role of oncogenic KRAS in the diagnosis, prognosis and treatment of pancreatic cancer. Nat. Rev. Gastroenterol. Hepatol..

[33] Samstein RM (2019). Tumor mutational load predicts survival after immunotherapy across multiple cancer types. Nat. Genet..

[34] Tang L (2020). DCST1-AS1 promotes TGF-β-induced epithelial-mesenchymal transition and enhances chemoresistance in triple-negative breast cancer cells via ANXA1. Front. Oncol..

[35] Zhang Y (2019). Long noncoding RNA TP53TG1 promotes pancreatic ductal adenocarcinoma development by acting as a molecular sponge of microRNA-96. Cancer Sci..

[36] Yang B, Gu B, Zhang J, Xu L, Sun Y (2020). CASC8 lncRNA promotes the proliferation of retinoblastoma cells through downregulating miR34a methylation. Cancer Manag. Res..

[37] Li Y (2020). LncRNA SNAI3-AS1 promotes PEG10-mediated proliferation and metastasis via decoying of miR-27a-3p and miR-34a-5p in hepatocellular carcinoma. Cell Death Dis..

[38] Oba A (2022). Prognosis based definition of resectability in pancreatic cancer: a road map to new guidelines. Ann. Surg..

[39] Peng F (2021). Development and validation of a nomogram to predict survival in pancreatic head ductal adenocarcinoma after pancreaticoduodenectomy. Front. Oncol..

[40] Winer LK (2019). The impact of tumor location on resection and survival for pancreatic ductal adenocarcinoma. J. Surg. Res..

[41] Sung MK (2021). Comparison of characteristics and survival rates of resectable pancreatic ductal adenocarcinoma according to tumor location. Biomedicines..

[42] Malleo G (2020). Does site matter? Impact of tumor location on pathologic characteristics, recurrence, and survival of resected pancreatic ductal adenocarcinoma. Ann. Surg. Oncol..

