# Supplementary material for: Prognostic signature based on m6A-related lncRNAs to predict overall survival in pancreatic ductal adenocarcinoma
Source: Sci Rep. 2022 Feb 23;12:3079. doi: 10.1038/s41598-022-07112-8 (PMC8866422; doi:10.1038/s41598-022-07112-8)
Supplement: Supplementary file 1 — Supplementary Information 1. [file 41598_2022_7112_MOESM1_ESM.pdf]

Supplementary Figures

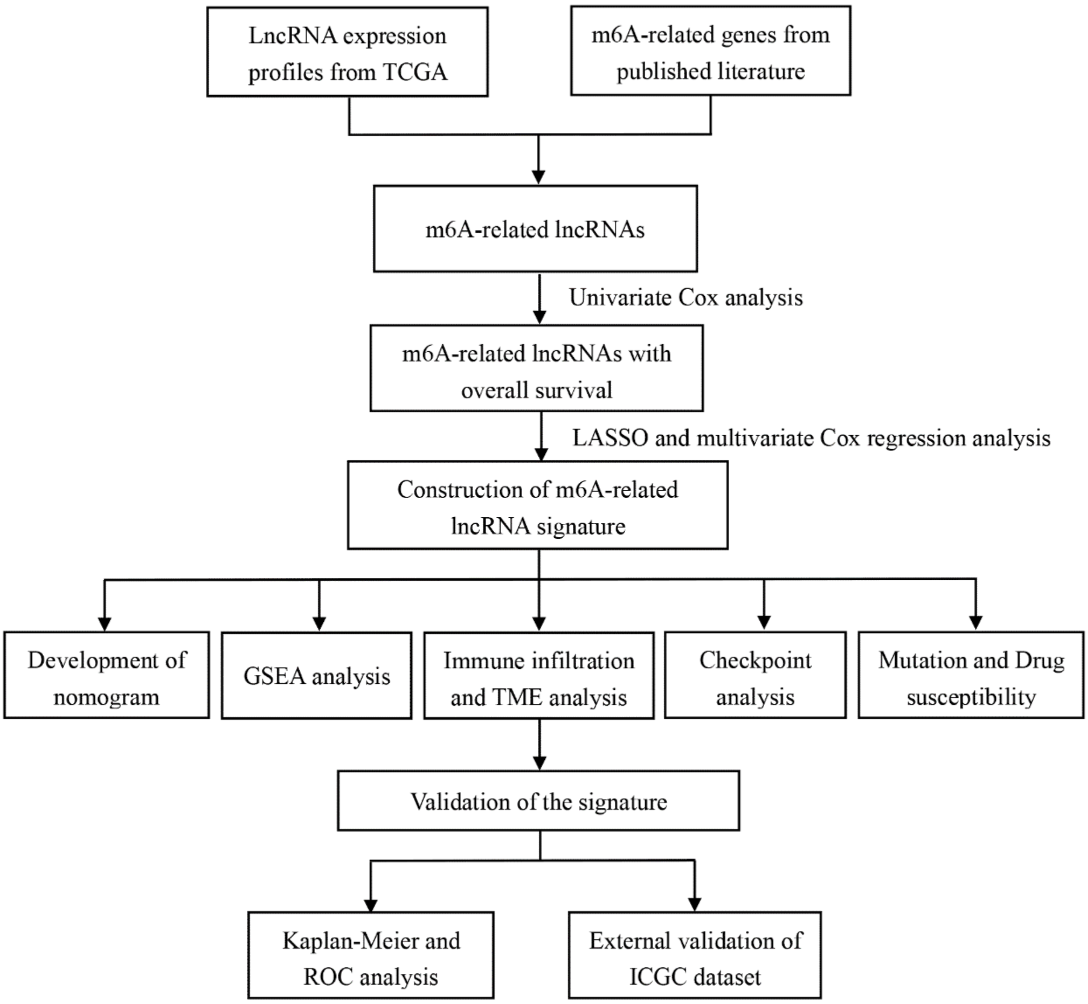

Supplementary Figure 1 The flow chart of data analysis in this study.

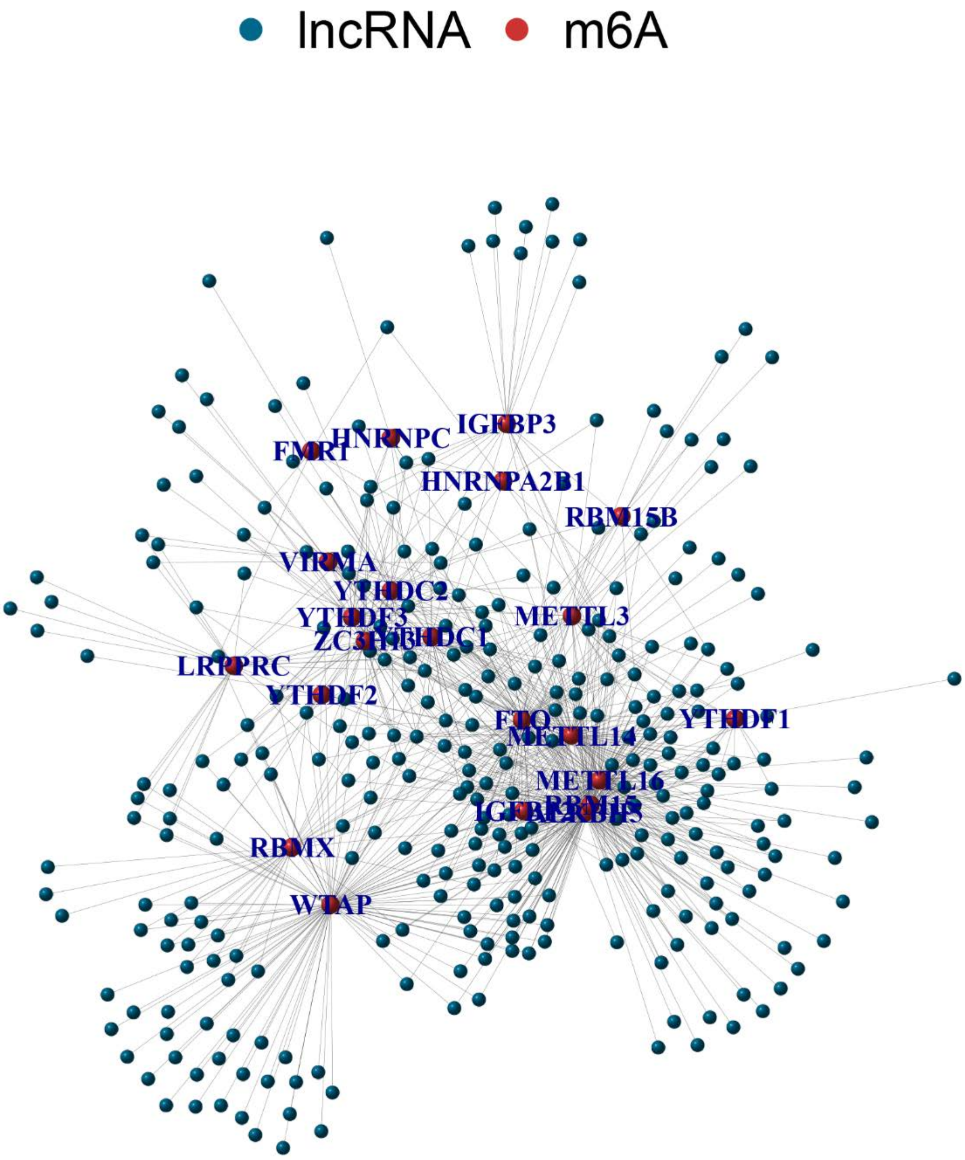

Supplementary Figure 2 The network of the m6A-related regulators and lncRNAs.

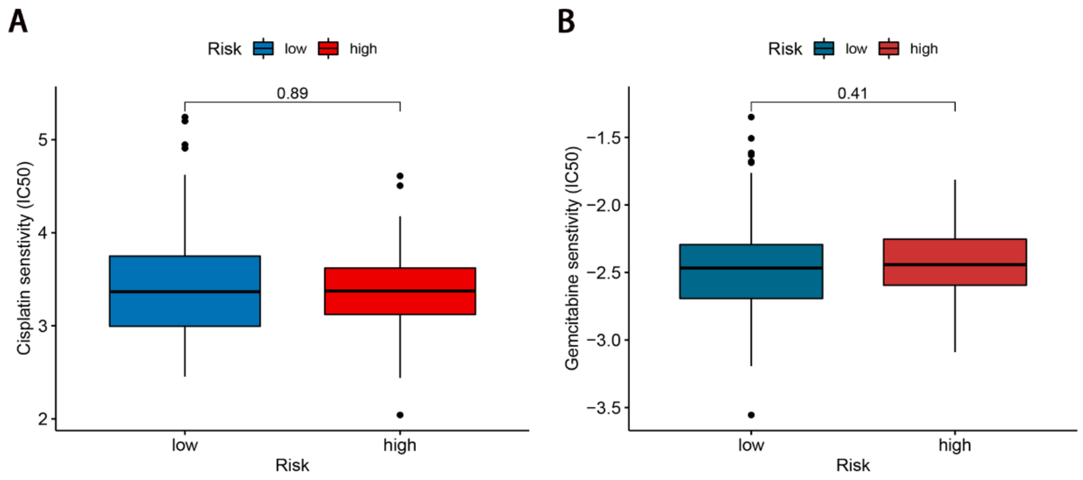

Supplementary Figure 3 Comparison of drug sensitivity analyses between the two risk groups.

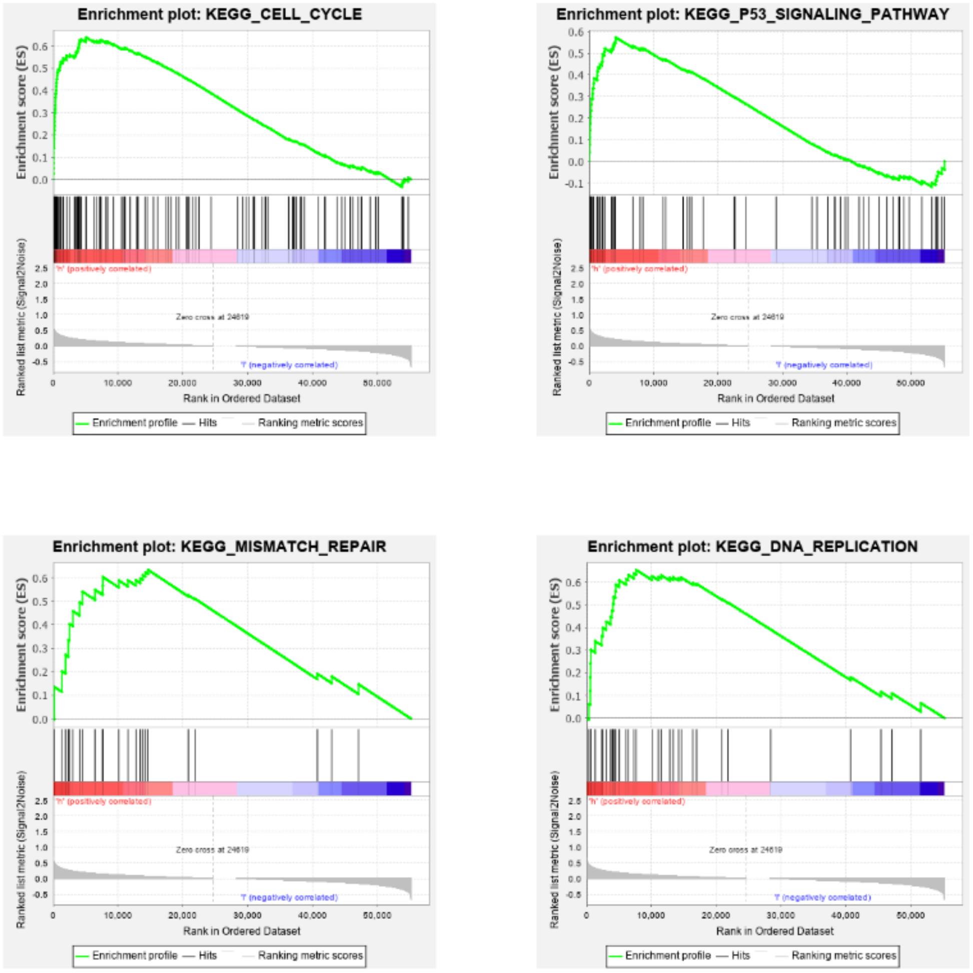

Supplementary Figure 4 Gene set enrichment analysis of two risk groups.
